# Constructing Equity Investment Strategies Using Analyst Reports and Regime Switching Models

**DOI:** 10.3389/frai.2022.865950

**Published:** 2022-05-18

**Authors:** Rei Taguchi, Hikaru Watanabe, Hiroki Sakaji, Kiyoshi Izumi, Kenji Hiramatsu

**Affiliations:** ^1^School of Engineering, The University of Tokyo, Tokyo, Japan; ^2^Faculty of Engineering, The University of Tokyo, Tokyo, Japan; ^3^IFIS Japan Limited, Tokyo, Japan

**Keywords:** BERT, Hidden Markov Model, trading strategy, financial market, regime switching model

## Abstract

This study demonstrates whether analysts' sentiments toward individual stocks are useful for stock investment strategies. This is achieved by using natural language processing to create a polarity index from textual information in analyst reports. In this study, we performed time series forecasting for the created polarity index using deep learning, and clustered the forecasted values by volatility using a regime switching model. In addition, we constructed a portfolio from stock data and rebalanced it at each change point of the regime. Consequently, the investment strategy proposed in this study outperforms the benchmark portfolio in terms of returns. This suggests that the polarity index is useful for constructing stock investment strategies.

## 1. Introduction

In recent years, the explosive development of artificial intelligence has brought worldwide attention to the use of alternative data, which is particularly prominent in the financial and economic fields and is beginning to be widely used for economic forecasting and investment strategies together with traditional data. In Japan, the Japan Association for the Promotion of Alternative Data (JADAA)^1^ and other cross-industry organizations have been established to promote its use actively.

This study is expected to expand the application possibilities of alternative data. Additionally, this study aims to encourage the expansion of artificial intelligence techniques in investment technology by providing the same reliability criteria as existing economic statistics in this area using indexes produced from alternative data. Among them, text data is particularly versatile and can extract predictions and classifications based on the writing style of the author and the content bais (Izumi and Sakaji, 2019; Sakaji et al., 2019).

Moreover, this study is expected to expand the possibilities of data linkage. The purpose of data linkage in this study is to encourage the expansion of artificial intelligence technology in the investment field by providing the same reliability criteria as existing statistical information in the investment field through alternative data analysis.

In addition, analyst reports are considered to have a very high information value among text data (Hiramatsu et al., 2021). Analyst reports contain rating information on individual stocks; therefore, the polarity index generated from these reports is assumed to affect stock prices, Return on Equity and Price Earnings Ratio. The polarity index is a time series plot of the polarity assigned to the text data. In recent years, it has been found that polarity index generated from analyst reports are ahead of macroeconomic indexes such as exchange rates and government bonds issuance (Taguchi et al., 2021) and are expected to be applied to investment technology.

Although there are many studies on portfolio management methods using machine learning (Wang et al., 2020; Yun et al., 2020; Zhang et al., 2020; Chen et al., 2021; Ma et al., 2021), this study is novel as we utilize the method of sentiment analysis into portfolio management.

This study shows that sentiment toward the future of individual stocks in analyst reports is useful in investment. The expected outcome is that the investment strategy proposed in this study will generate returns exceeding the benchmark portfolio. Consequently, it is envisioned that changes in the regime of polar indicators generated from analyst reports will serve as signals to change the investment allocation of individual stocks.

This study's contribution is to demonstrate that an investment strategy can generate returns by using analysts' sentiments toward individual stocks to signal portfolio asset allocation. From this, we demonstrate that analyst reports are useful for investment.

## 2. Related Works

There is considerable literature on portfolio management methods using machine learning and deep learning. Ma et al. (2021) performed return prediction with several opportunity learning models, including deep multilayer perceptron, and incorporated the prediction results into the advanced mean-variance model for portfolio optimization. Yun et al. (2020) proposed a two-stage deep learning model for a forecast-based Exchange Traded Fund (ETF) portfolio management approach. Wang et al. (2020) proposed a portfolio management method that considers the long-term dependence of time series variability using mean-variance models and deep learning theories such as Long Short Term Memory (LSTM). Zhang et al. (2020) proposed a deep learning framework for maximizing the Sharpe ratio of a portfolio. Chen et al. (2021) proposed a new model to optimize XGBoost with an improved firefly algorithm (IFA) and perform portfolio optimization.

Some studies have been conducted on creating polarity indicators by machine learning. In Yono et al. (2020), Financial and Economic Statistics Monthly were trained using LSTM, and indicators for each topic such as consumer spending, capital investment, and inventory were created using Latent Dirichlet Allocation (LDA). They also analyzed which macro factors are more influential by determining how much each topic's sentiment contributes to the overall sentiment. Katayama et al. (2019) developed a sentiment polarity identification model for finance by processing the Japan Economic Watcher Survey with Word2vec and training it with LSTM.

The above studies use texts with similar contents, such as the Financial and Economic Statistics Monthly and the Economic Watcher Survey. In contrast, our study differs in that we use analyst reports as an indicator by summing up the sentiment of individual stocks. Some studies have been conducted as an example of research on text mining using analyst reports. In Hiramatsu et al. (2021), stock prices respond strongly to the sentiment of the report, and drift in stock prices is observed after the report is issued, suggesting that the textual information in analyst reports is useful in asset management practice. Further, Asquith et al. (2005) investigates the association between market returns and analyst reports content using regression analysis. In addition, Suzuki et al. (2020) opinion and non-opinion text are extracted from analyst reports using LSTM and other methods, and forecasts of net income and stock prices are made.

Our study's use of Bidirectional Encoder Representations from Transformers (BERT) to train analyst reports is also different from other studies. BERT is a Transformer-based language model. An example of a study using BERT is Hiew et al. (2019) where text data is given polarity by BERT using Weibo, a Chinese Social Networking Service, and stock price predictions are made using LSTM.

Furthermore, our study differs from other studies in that we use Bi-directional Gated Recurrent Unit (BiGRU) and Hidden Markov Model with Gaussian Mixture Model emissions (GMM-HMM) to trigger the portfolio rebalancing. An example of a study using BiGRU in the financial field is Chen et al. (2020) which used labeled financial tweet data and BiGRU to perform sentiment analysis. Liu et al. (2021) used GMM-HMM and LSTM to predict stock prices. BiGRU and LSTM have several applications outside the financial sector (Talha et al., 2017; Yan et al., 2021).

## 3. Methods

In this study, we performed asset allocation for a stock portfolio composed of two stocks, using signals as change points in the polar indicators regime created from analyst reports. We used analyst reports to develop an investment strategy using natural language processing and artificial intelligence techniques in the following four steps. We demonstrate that analyst reports are useful for investment by comparing this investment strategy with the benchmark strategy.

**Step 1:** BERT is used to learn the analyst report text and classify the polarity into three values: “positive,” “negative,” and “neutral.” A detailed description of the method is given in section 3.1, and the results are shown in section 5. This step aims to create a polarity index.

**Step 2:** We perform time series forecasting for the created polarity index (by industry) using a neural network. The algorithm used is Bi-directional Gated Recurrent Unit (BiGRU). A detailed description of the method is given in section 3.2, and the results are shown in section 5. This step in forecasting polar indicators aims to make a preliminary step in creating signals for future asset allocations.

**Step 3:** A three-month moving average is taken for the forecast value calculated in Step 2, and the volatility is clustered into “high,” “medium,” and “low” states using the regime-switching model. The algorithm used is the Hidden Markov Model with GMM-HMM. A detailed description of the method is provided in section 3.3 and the results are presented in section 5. This step creates a signal for asset allocation at a future point that has not yet manifested by dividing the polarity index predicted in Step 2 into three regimes.

**Step 4:** We retrieved monthly stock price data from Yahoo! Finance^2^. We selected two stocks for each of the 33 industries and created a portfolio for each industry using the model selected in Steps 2 and 3. Then we rebalanced the portfolio at the regime's turn assigned in Step 3 above. In addition, we compare this strategy with the benchmark strategy and tabulate the results. A detailed description of the method is given in section 3.4, and the results are shown in section 5.

**Figure 1:**
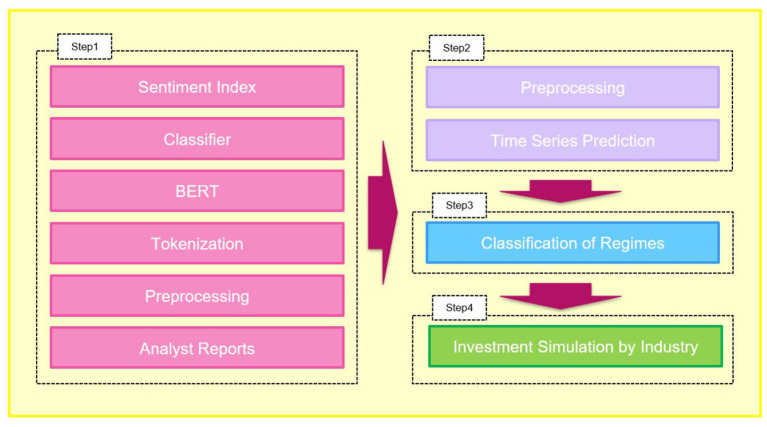
Architecture.

The architecture used in this study can be described as shown in Figure 1.

We created a polarity index as discussed. The assumption is that the sentiment of individual stocks is included in this polarity index, which is used for the investment experiment.

### 3.1. Bidirectional Encoder Representations From Transformers (BERT)

BERT is a Transformer-based language model proposed by Devlin et al. (2019). The reason we chose BERT for this study is because it has demonstrated its effectiveness against other models in eight benchmarks in Devlin et al. (2019). BERT enables successful bidirectional learning by masking some tokens in the input and predicting the masked words. This study used the model^3^ published by Inui and Suzuki Laboratory at Tohoku University as a trained model for Japanese BERT. Additionally, we use the method of Taguchi et al. (2021) to create two types of models (UP Model and DOWN Model) using BERT. In the Taguchi et al. (2021) method, a report with a change in rating to “buy” is considered to be 1, and 0 otherwise, and a report with a change in rating to “sell” is considered to be −1. The final judgment method is shown in Table 1. Details of this method can be obtained by referring to Taguchi et al. (2021). In addition, this architecture replaces the model in Sakaji et al. (2008) with BERT. See section 5 for the calculation results.

**Table 1:** Polarity judgment method.

**UP model**	**DOWN model**	**Final judgment**
0 (no change)	−1 (sell)	−1 (sell)
1 (buy)	0 (no change)	1 (buy)
1 (buy)	−1 (sell)	High likelihood
0 (no change)	0 (no change)	0 (no change)

### 3.2. Time Series Forecasting Using Bi-Directional Gated Recurrent Unit (BiGRU)

Section 3.2 aims to take a preliminary step in creating a signal for future asset allocation. Time series forecasting creates pseudo-sentiment information not apparent. Gated Recurrent Unit (GRU) is a neural network consisting of Reset and Update gates, proposed by Cho et al. (2014). GRU is a neural network that can deal with the gradient vanishing problem of Recurrent Neural Network (RNN) and has a low computational cost. This point was corroborated by the verification of performance in section 5.

**Figure 2:**
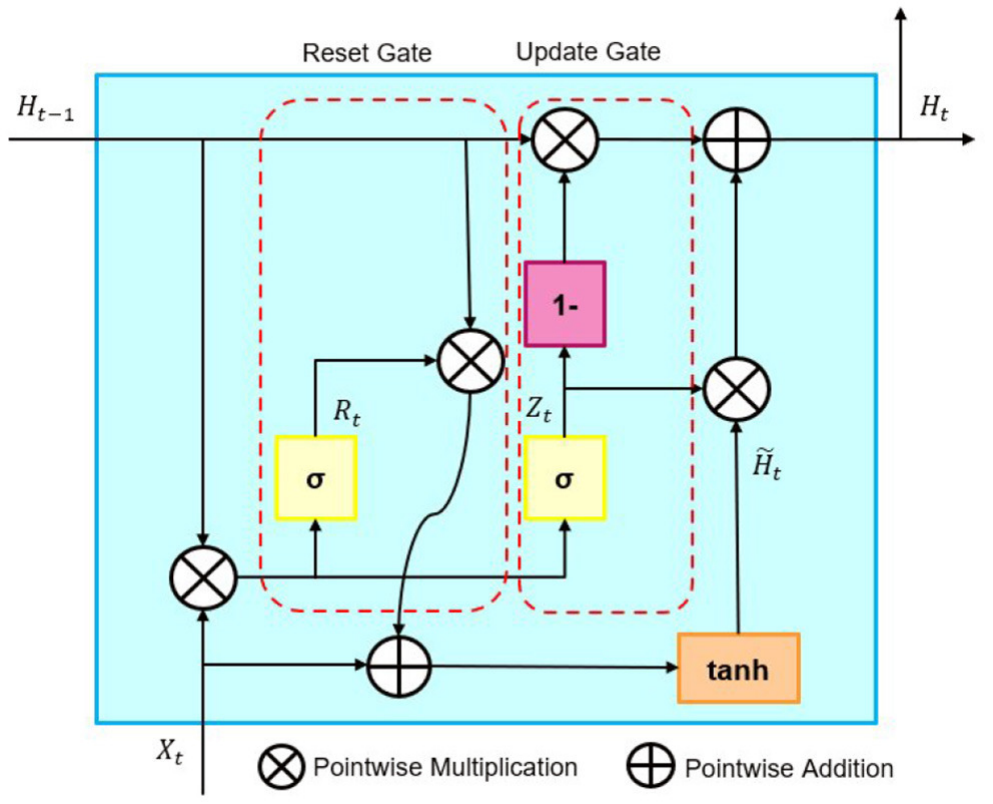
Architecture of GRU.

The architecture of GRU is represented by Figure 2. BiGRU is a model that can add past and future information to GRU.

**Figure 3:**
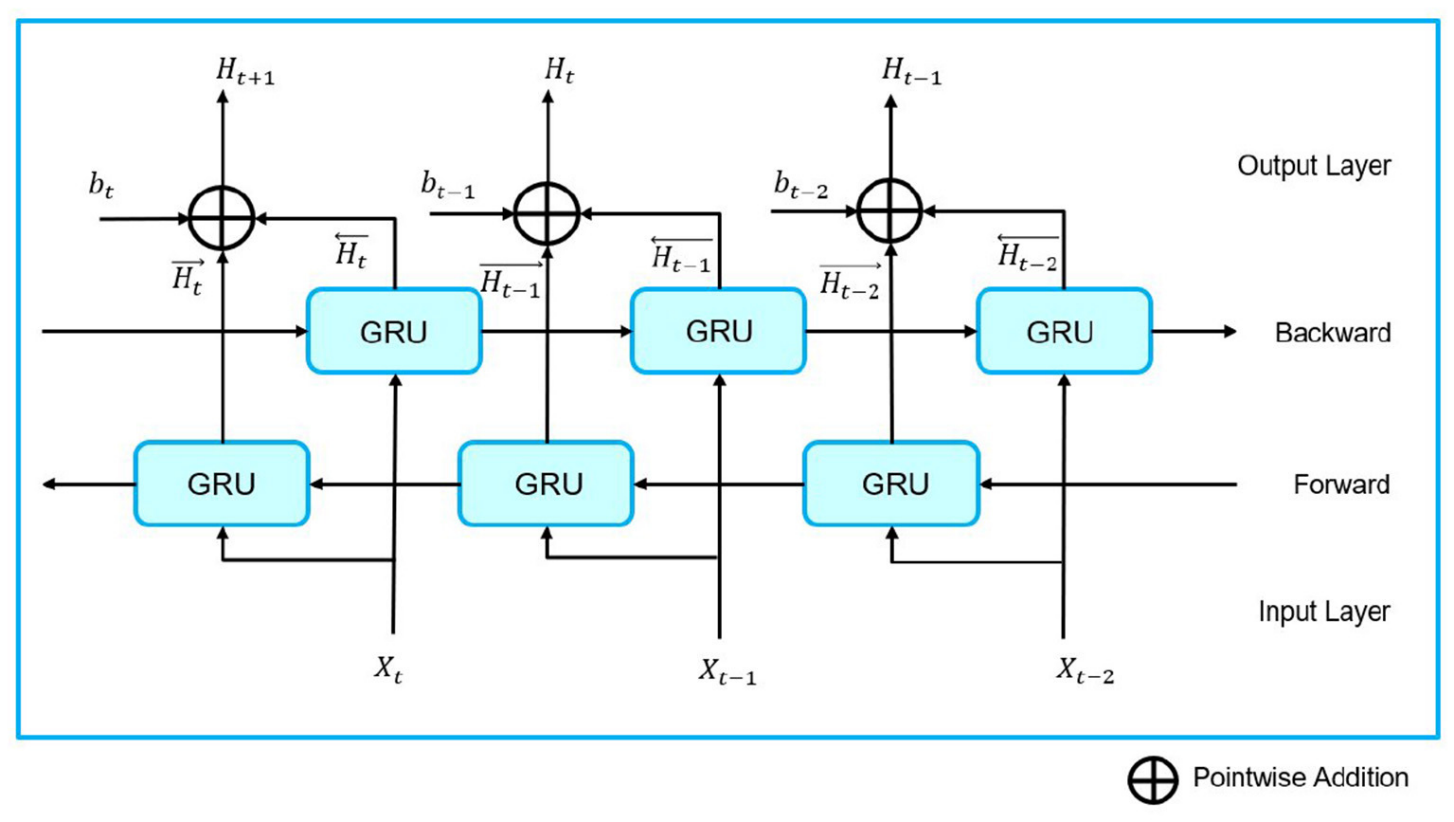
Architecture of BiGRU.

The architecture of BiGRU is represented by Figure 3. Details of the GRU and BiGRU algorithms are given in Appendix A.

Time series forecasting was evaluated using Root Mean Squared Error (RMSE). RMSE is a measure to evaluate the model's error, and the smaller it is, the better the model's performance. The equation for RMSE is as follows.


(1) $$\begin{array}{cc} \text{RMSE} & =\sqrt{\frac{1}{n}\sum_{i=1}^{n}{\left(y_{i}-\hat{y_{i}}\right)}^{2}} \end{array}$$

RMSE is calculated by dividing the observed values by *y*_*i*_(*i* = 1, 2, 3, …, *n*), and the calculated value (predicted value) from the model is ŷ_*i*_.

### 3.3. Regime Assignment Using Hidden Markov Model With Gaussian Mixture Model Emissions (GMM-HMM)

In section 3.3, we divide the polarity index predicted in section 3.2 into three regimes to create a signal for asset allocation in the future not yet manifested. The changes in the regimes are assumed changes in the environment and are used as signals.

**Figure 4:**
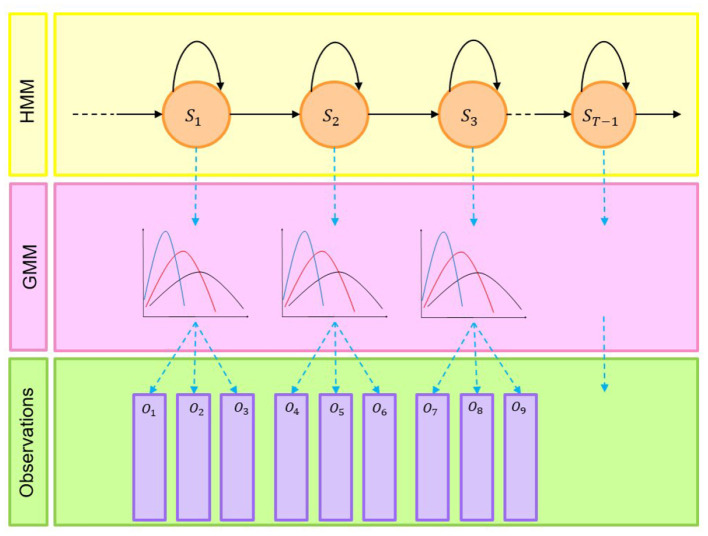
Architecture of GMM-HMM.

This study used the Hidden Markov Model (HMM) with the Gaussian Mixture Model (GMM) state output distribution to assign regimes to the predicted values of the polarity index. GMM is a statistical model that can model N populations following a normal distribution, while HMM is a Markov model based on a Markov process with hidden states. The architecture of GMM-HMM is represented by Figure 4.

Details of the GMM-HMM algorithms are given in Appendix B.

### 3.4. Investment Simulation

In this section, we design an investment simulation using the models in sections 3.2 and 3.3. As a proposed method, when the volatility of the polarity index predicted by the GMM-HMM with three regimes is “low,” we increase the allocation to stocks with high monthly volatility. In addition, when the predicted volatility of the polarity index is “medium,” the investment allocation is set at 50%. Meanwhile, if the forecasted volatility of the polarity index is “high,” we increase the allocation to stocks with low monthly volatility. The monthly volatility of a stock is defined as the maximum value among the current month's True Range^4^ ÷Typical Price for the current month^5^ × 100, which is calculated below. We assume that the above portfolio rebalancing strategy generates higher returns than the benchmark strategy.

See Table 2 for Notation in the investment simulation. The total profit and loss of the portfolio *P* are defined below. We assume that the portfolio consists of two stocks types.


(2) $$\begin{array}{rc} P & =P_{L}+P_{N}+P_{H} \end{array}$$

*P*_*L*_ is the sum of the portfolio's profit and loss when the polarity index volatility is “low,” *P*_*N*_ is the sum of the portfolio's profit and loss when the polarity index volatility is “medium,” and *P*_*H*_ is the sum of the portfolio's profit and loss when the polarity index volatility is “high.” The total of each profit and loss can be formulated as follows.


(3) $$\begin{array}{l} \begin{array}{cc} P_{L} & =Am*\sum_{n=1}^{NoR}\left\{(1-r_{n})*(S_{n}^{L}-B_{n}^{L})+r_{n}*(S_{n}^{H}-B_{n}^{H})\right\}, \end{array}\\ \;\begin{array}{c} \text{if }RoPI\text{ is 0} \end{array} \end{array}$$

(4) $$\begin{array}{l} \begin{array}{cc} P_{N} & =Am*\sum_{n=1}^{NoR}\{0.5*(S_{n}^{L}-B_{n}^{L})+0.5*(S_{n}^{H}-B_{n}^{H})\}, \end{array}\\ \;\begin{array}{c} \text{if }RoPI\text{ is 1} \end{array} \end{array}$$

(5) $$\begin{array}{l} \begin{array}{cc} P_{H} & =Am*\sum_{n=1}^{NoR}\{r_{n}*(S_{n}^{L}-B_{n}^{L})+(1-r_{n})*(S_{n}^{H}-B_{n}^{H})\}, \end{array}\\ \;\begin{array}{c} \text{if }RoPI\text{ is 2} \end{array} \end{array}$$

Conversely, the total profit and loss of the benchmark strategy to be compared, *P*_*BM*_, is the profit and loss of the two stocks purchased at the beginning of the experiment with the investment ratio of *r* = 0.5 and sold at the end of the experiment period. *P*_*BM*_ is formulated as follows:


(6) $$\begin{array}{r} P_{BM}=Am*\left\{0.5*\left(S_{\alpha }-B_{\alpha }\right)+0.5*\left(S_{\beta }-B_{\beta }\right)\right\} \end{array}$$

In comparing the benchmark and proposed strategies, we assume that both portfolios are composed of two similar stocks.

**Table 2:** Notation of investment simulations.

**Notation**	
*RoPI*	Regime of polarity index (volatility low = 0, medium = 1, high = 2)
*Am*	Amount of stock purchased
*NoR*	Number of rebalancing (1, …, *n*)
*r*	Investment ratio (0 ≤ *r* ≤ 1)
*B* ^*L*^	Buying price for stocks with “low” volatility
*S* ^*L*^	Selling price for stocks with “low” volatility
*B* ^*H*^	Buying price for stocks with “high” volatility
*S* ^*H*^	Selling price for stocks with “high” volatility
*B*_α_	Buying price at the begin of the investment simulation for α
*S*_α_	Selling price at the end of the investment simulation for α
*B*_β_	Buying price at the begin of the investment simulation for β
*S*_β_	Selling price at the end of the investment simulation for β

**Figure 5:**
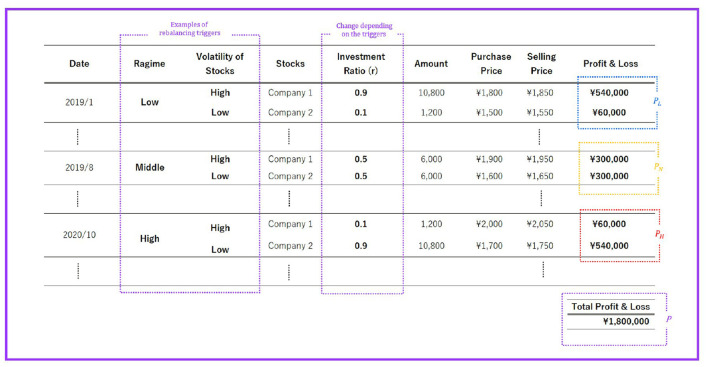
Example of investment simulations.

The overall picture of the investment simulation is shown in Figure 5. In Figure 5, we set the investment ratio *r* = 0.9. For the results of the investment simulation, see section 5.

## 4. Experiments

Analyst report data (373,050) was used to create the BERT polarity index. Utilizing the technologies of IFIS Japan Ltd, charts and unnecessary texts in research reports were removed. The data period is from Jan 2010 to December 2020. Of the data used in BERT, the train, valid, and test ratios are 7:1:2, respectively. In this study, five models were used for time series forecasting. The first is Bi-directional GRU (BiGRU); the second is GRU; the third is Bi-directional LSTM (BiLSTM); the fourth is Recurrent Neural Network (RNN), and the fifth is LSTM. We take three-month moving averages for the all-industry polarity index in preprocessing the data. We use monthly stock price data obtained from Yahoo! Finance for the investment simulation. See Appendix C for the stocks used in the experiments.

## 5. Results

We created a polarity index for all industries and each industry. The all-industry polarity index was used for model selection, and the industry-specific polarity index for investment simulation. The all-industry polarity index is the sum of all outputs (1, 0, −1) of the industry-specific polarity index.

**Figure 6:**
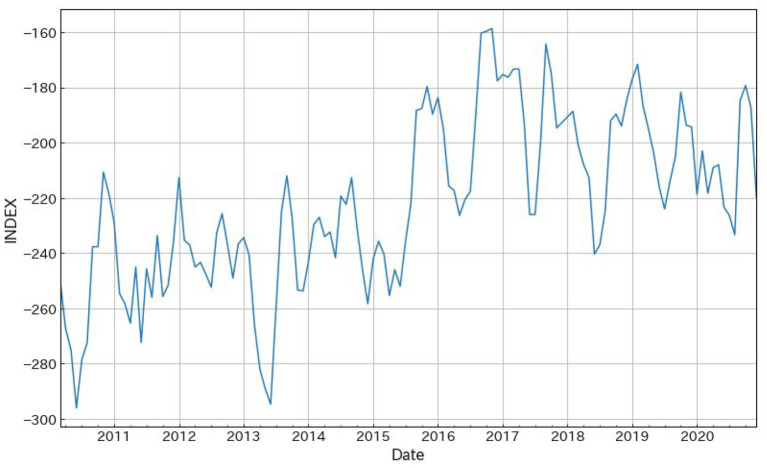
All-industry polarity index with 3-month moving average.

The 3-month moving averages of the all-industry polarity index, sorted by time series, are shown in Figure 6.

The results of the comparison experiments are shown in Table 3. We chose BiGRU, the smallest RMSE among the five in this study.

**Table 3:** Time series prediction.

**Model**	**RMSE**
BiGRU	0.1376
GRU	0.1600
BiLSTM	0.2011
RNN	0.2115
LSTM	0.2156

In this study, we used three models for clustering. The first is GMM-HMM, the second is Gaussian Hidden Markov Model (Gaussian-HMM), and the third is Markov witching Dynamic Regression Models (MS-DRM). In pre-processing the data, three-month moving averages are taken for the predicted values of the all-industry polarity index. The log-likelihood was used as the evaluation index; the larger the model's log-likelihood, the better the model's fit.

The results of the comparison experiments are shown in Table 4. We chose the GMM-HMM with the largest log-likelihood among the three in this study.

**Table 4:** Assigning regimes.

**Model**	**Log likelihood**
GMM-HMM	−72.3536
Gaussian-HMM	−72.3544
MS-DRM	−84.0420

**Figure 7:**
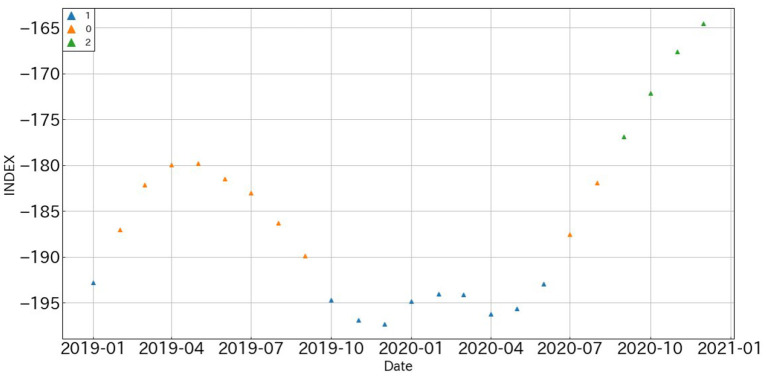
Predicted values for all-industry polarity index with regimes.

The results of assigning regimes to the predictions of the polarity indexes using GMM-HMM are shown in Figure 7.

The investment simulation results in section 3.4 are shown. Monthly stock price data is obtained from Yahoo! Finance. For details of the investment simulation, see section 3.4. This experiment assumes that the amount of stocks *Am* = 12, 000. See Appendix C for the constituent stocks of each portfolio.

The investment simulation results are shown in Table 5. In the table, ° refers to the victory of the proposed strategy against the benchmark, and • refers to the defeat of the proposed strategy against the benchmark. The *NoR* in the table refers to the number of times the 33-industry portfolio is rebalanced.

**Table 5:** Winners and losers of proposed strategies in benchmarking strategies.

	**Investment ratio (r)**									

**Industry name**	**NoR**	***r*** **= 0**	***r*** **= 1**	***r*** **= 2**	***r*** **= 3**	***r*** **= 4**	***r*** **= 5**	***r*** **= 6**	***r*** **= 7**	***r*** **= 8**	***r*** **= 9**	***r*** **= 10**
Fisheries, Agriculture and Forestry	10	•	•	•	°	°	°	°	°	°	°	°
Mining	5	•	•	•	°	°	°	°	°	°	°	°
Construction	5	•	•	•	•	•	•	°	°	°	°	°
Foodstuffs	8	°	°	°	°	°	°	°	°	°	°	°
Textiles	7	•	•	•	•	•	•	•	•	•	•	•
Pulp and Paper	5	°	°	°	°	°	°	°	°	°	°	°
Chemistry	5	•	•	•	•	°	°	°	°	°	°	°
Pharmaceuticals	4	•	•	•	•	•	•	•	•	°	°	°
Oil and Coal Products	3	•	•	•	•	•	•	•	•	•	•	•
Rubber Products	6	°	°	°	°	°	°	°	°	°	°	°
Glass, Clay and Stone Products	5	°	°	°	°	°	•	•	•	•	•	•
Steel	5	°	°	°	°	°	°	•	•	•	•	•
Nonferrous Metals	4	•	•	•	•	•	•	•	•	•	•	•
Metal Products	5	•	•	•	•	•	•	•	•	°	°	°
Machinery	7	°	°	°	°	°	°	°	°	°	°	°
Electronic Equipment	3	°	°	°	°	°	•	•	•	•	•	•
Transport Equipment	7	•	•	•	•	•	•	•	•	•	•	•
Precision Equipment	4	•	•	•	•	•	•	•	•	•	•	•
Other Products	8	•	•	•	•	•	•	•	°	°	°	°
Electricity and Gas	4	°	•	•	•	•	•	•	•	•	•	•
Land Transportation	3	•	•	•	•	•	•	°	°	°	°	°
Marine Transportation	6	•	•	•	•	•	•	•	•	•	•	•
Air Transportation	5	•	•	•	•	•	•	•	•	•	•	•
Warehousing and Transportation	5	°	•	•	•	•	•	•	•	•	•	•
Information and Communication	4	•	•	•	•	°	°	°	°	°	°	°
Wholesale business	6	•	•	•	•	•	•	°	°	°	°	°
Retailing	6	•	•	•	•	•	•	•	•	•	•	•
Banking	7	•	•	•	•	•	•	•	•	•	°	°
Securities and Commodity Futures	10	•	•	•	•	•	•	•	•	•	•	•
Insurance	5	°	°	°	°	°	°	°	°	°	°	•
Other Financial Services	5	•	•	•	•	•	•	•	•	•	•	•
Real Estate	7	•	•	•	•	•	•	•	•	•	•	•
Service	7	°	°	°	°	•	•	•	•	•	•	•

## 6. Discussion

First, Table 5 reveals that the $\frac{15}{33}$ industry wins when the investment ratio is *r* = 0.8, and the $\frac{16}{33}$ industry wins when *r* = 0.9. This indicates that the strategy of increasing the allocation to stocks with high monthly stock volatility when the predicted values volatility of the polarity index is “low,” setting the investment allocation to 50% when the predicted values volatility of the polarity index is “medium,” increasing the allocation to stocks with low monthly stock volatility when the predicted values volatility of polarity index is “high,” is somewhat effective against the benchmark strategy.

Second, among the portfolios of 33 industries, we observed seven industries in which the profit and loss of the benchmark strategy were negative. These include pulp and paper, glass and stone products, and electric equipment. For these industries, even when the investment ratio *r* was changed, many of them showed extreme trends such as winning all patterns and losing all patterns. In some cases, such as glass and stone products and electrical equipment, the results contradict our hypothesis. Although it is impossible to determine whether the tendency is industry-specific or market-influenced only from the results of this experiment, we would like to explore a method to determine the investment ratio based on these results when constructing a portfolio among industries.

Third, we found that the number of *NoR* varies significantly among industries. For example, the number of *NoR* in the fisheries, agriculture, and forestry industry is ten, whereas the land transportation industry is three. This indicates that the tendency to change the volatility of polar indexes is remarkably different among industries.

Fourth, in Table 3, we expected BiLSTM to have the lowest RMSE because its mechanism is richer among the five; however, BiGRU has the lowest RMSE. Chung et al. (2014) shows that the superiority or inferiority of LSTM and GRU cannot be determined in general but depends on the data set and the given task. The same can be said for BiLSTM, LSTM, BiGRU, and GRU in this experiment.

Fifth, there is still room for improvement in the duration of signal creation, as shown in the GMM-HMM results (Table 4). Since, in practice, the asset allocation of portfolios is often done monthly, we used monthly data for both text and stocks. It is necessary to use frequent data to create more detailed signals.

Sixth, although the investment experiment in this study was conducted with two stocks, there are many cases where three or more stocks are used in actual operations. In future research, we would like to extend the investment model to multiple dimensions and give priority to experiments with three or more stocks. The following points are specific implementation methods for portfolios of three or more stocks. First, we need to ensure the diversification effect of stocks by using correlation coefficients. Second, as the number of stocks increases, the timing of rebalancing increases in proportion to the number of stocks, it is necessary to obtain higher frequency data on text and stock price data. It is also important to set a quantitative threshold for the volatility of stocks. In addition, it is desirable to use methods such as the mean-variance model proposed by Ma et al. (2021) and Wang et al. (2020) when determining the maximum investment ratio.

Finally, although the experiments in this study were limited to stock investment strategies, we would like to extend this study to future corporate bonds, Foreign Exchange and virtual currencies.

## 7. Conclusion

In this study, we created a polarity index using BERT, performed time series forecasting using BiGRU, and assigned a regime to the forecasted values using GMM-HMM to create a signal for portfolio rebalancing. Consequently, the strategy proposed in this study proved effective, indicating that analyst reports are useful for investment. In the future, we would like to create a signal that replaces the architecture of the regime-switching model with an anomaly detection algorithm. In addition, we would like to conduct investment experiments with three or more stocks in one portfolio since we have created two stocks in one portfolio in this experiment. We seek to calculate the investment ratio using reinforcement learning in conjunction with this. Furthermore, we would like to conduct investment simulations using polar indexes created by other financial texts and compare the current results.

## Data Availability Statement

The data analyzed in this study is subject to the following licenses/restrictions: this data can be used for academic authorized researchers. Requests to access these datasets should be directed to KH, kenji.hiramatsu@ifis.co.jp; HS, sakaji@sys.t.u-tokyo.ac.jp.

## Author Contributions

RT, HW, HS, and KI contributed to the conception and design of the study. KH organized the database. RT and HW performed the statistical analysis. RT wrote the first draft of the manuscript and sections of the manuscript. All authors contributed to the revision of the manuscript and read and approved the submitted versions.

## Funding

This study was partially supported by JST-Mirai Program Grant Number JPMJPI20B1, Japan. This study was not funded by IFIS Japan Limited.

## Conflict of Interest

KH is employed by IFIS Japan Limited. The remaining authors declare that the research was conducted in the absence of any commercial or financial relationships that could be construed as a potential conflict of interest.

## Publisher's Note

All claims expressed in this article are solely those of the authors and do not necessarily represent those of their affiliated organizations, or those of the publisher, the editors and the reviewers. Any product that may be evaluated in this article, or claim that may be made by its manufacturer, is not guaranteed or endorsed by the publisher.

## Footnotes

^1^ https://alternativedata.or.jp

^2^ https://finance.yahoo.co.jp/

^3^ https://github.com/cl-tohoku/bert-japanese

^4^Defined in this article as high-low, high-close, and close-low.

^5^Average of the high, low, and close prices.
