# Supplementary material for: Constructing Equity Investment Strategies Using Analyst Reports and Regime Switching Models
Source: Front Artif Intell. 2022 May 18;5:865950. doi: 10.3389/frai.2022.865950 (PMC9157435; doi:10.3389/frai.2022.865950)
Supplement: Supplementary file 1 [file Data_Sheet_1.pdf]

Constructing Equity Investment Strategies using Analyst Reports and Regime Switching Models

Rei Taguchi [1,*], Hikaru Watanabe [2], Hiroki Sakaji [1], Kiyoshi Izumi [1] and Kenji Hiramatsu [3]

[1] *School of Engineering, The University of Tokyo, Tokyo, Japan*
[2] *Faculty of Engineering, The University of Tokyo, Tokyo, Japan*
[3] *IFIS Japan Limited, Tokyo, Japan*

Correspondence*:
Rei Taguchi
s5abadiee@g.ecc.u-tokyo.ac.jp

REFERENCES

Baum, L. E., Petrie, T., Soules, G., and Weiss, N. (1970). A maximization technique occurring in the statistical analysis of probabilistic functions of markov chains. *The annals of mathematical statistics* 41, 164–171

Viterbi, A. (1967). Error bounds for convolutional codes and an asymptotically optimum decoding algorithm. *IEEE Transactions on Information Theory* 13, 260–269. doi:10.1109/TIT.1967.1054010

APPENDIX A ALGORITHM OF BIGRU

Details of the algorithm of BiGRU are given in this appendix. First, we describe the GRU algorithm. If the number of hidden units is h, the input of the small batch at time t is $X_t \in \mathbb{R}^{n*d}$ (the number of samples is n and the number of inputs is d), and the hidden state at time $t-1$ is $X_{t-1} \in \mathbb{R}^{n*h}$. The reset gate vector R_t at time t is as follows:

$$R_t = \sigma(X_t W_{xr} + H_{t-1} W_{hr} + b_r) \tag{1}$$

The updated gate vector Z_t at time t is

$$Z_t = \sigma(X_t W_{xz} + H_{t-1} W_{hz} + b_z) \tag{2}$$

The activation candidate vector $\tilde{H}_t$ at time t is

$$\tilde{H}_t = \tanh(X_t W_{xh} + (R_t \circ H_{t-1}) W_{hh} + b_h) \tag{3}$$

The output vector H_t at time t is

$$H_t = (1 - Z_t) \circ H_{t-1} + Z_t \circ \tilde{H}_t \tag{4}$$

$\sigma(\cdot)$ is the sigmoid activation function, expressed as $\sigma(x) = \frac{1}{1+e^{-x}}$. Moreover, $\circ$ denotes the hadamard product. In addition, $\tanh$ is the hyperbolic tangent function, and expressed as $\tanh(x) = 1 - \frac{2}{1+e^{-2x}}$.

Second, we describe the BiGRU algorithm. The state H_t of the hidden layer at time t is determined from the input X_t at time t, the output $\overrightarrow{H_t}$ of the hidden layer in forward propagation, and the output $\overleftarrow{H_t}$ of the hidden layer in reverse propagation and can be formulated as follows:

$$\overrightarrow{H_t} = GRU(H_t, \overrightarrow{H_{t-1}}) \tag{5}$$

$$\overleftarrow{H_t} = GRU(H_t, \overleftarrow{H_{t-1}}) \tag{6}$$

$$H_t = w_t\overrightarrow{H_t} + v_t\overleftarrow{H_t} + b_t \tag{7}$$

The $GRU(\cdot)$ function applies a nonlinear transformation to the input data using GRU.

Table 1. Notation of GRU

Notation	
W_{xr}	weight vector for the input of the reset gate
W_{hr}	weight vector for the output of the reset gate
W_{xz}	weight vector for the input of the update gate
W_{hz}	weight vector for the output of the update gate
w_t	weight of the hidden layer output $\overrightarrow{H_t}$ in forward propagation
v_t	weight of the hidden layer output $\overleftarrow{H_t}$ in back propagation
b_r	bias of reset gate
b_z	bias of update gate
b_t	bias of hidden layer at time t

Please see Table 1 for GRU notation.

APPENDIX B ALGORITHM OF GMM-HMM

Details of the algorithm of GMM-HMM are presented in this appendix. In GMM-HMM, the hidden states are generated according to a Gaussian mixture distribution. In addition, in GMM-HMM, the output probability matrix B can be approximated as a combination of products of mixture Gaussian distributions.

- Determine the number N of hidden states.
- In the observation series O, calculate and record the parameters of the HMM $\lambda = (A, B, \pi)$ using the BaumWelh algorithm (Baum et al., 1970).
- The probability $P\{S_t = i\}, i = \{1, ..., N\}, t = \{0, ..., T-1\}$ is estimated using the Viterbi algorithm (Viterbi, 1967).

The process of the GMM-HMM algorithm is shown above.

Please see Table 2 for the Notation of GMM-HMM.

Table 2. Notation of GMM-HMM

Notation	
T	length of the observation sequence
N	number of states included in the model
M	number of observation symbols
A	transition probability matrix
B	output probability matrix
π	initial probability distribution vector
$O = \{O_0, ..., O_{T-1}\}$	observation sequence
$S = \{S_0, ..., S_{T-1}\}$	state sequence
$V = \{V_0, ..., V_{M-1}\}$	set of observable observations
$\lambda = (A, B, \pi)$	HMM

APPENDIX C COMBINATION OF STOCKS USED IN THE INVESTMENT SIMULATION

This table shows the combination of stocks used in the investment simulation in Section 4.4.

Table 3. Combination of Stocks used in the Investment Simulation

Industry Name	Constituent Stocks 1	Constituent Stocks 2
Fisheries, Agriculture and Forestry	Kyokuyo (1301)	Hokuto (1379)
Mining	Nittetsu Mining (1515)	Sumiseki HD (1514)
Construction	Tokyu Construction (1720)	UEKI Corporation (1867)
Foodstuffs	Morinaga Milk (2264)	DyDo Group HD (2590)
Textiles	Fujiboh HD (3104)	Katakura Industries (3001)
Pulp and Paper	Oji HD (3861)	Nippon Paper Group(3863)
Chemistry	Asahi Kasei (3407)	Nissan Chemical (4021)
Pharmaceuticals	Kyowa Kirin (4151)	Rohto Pharmaceutical (4527)
Oil and Coal Products	Yushiro Chemical Industry (5013)	Nippon Coke and Engineering (3315)
Rubber Products	Bridgestone Corporation (5108)	Sumitomo Rubber Industries (5110)
Glass, Clay and Stone Products	Nippon Electric Glass (5214)	Nittobo (3110)
Steel	Nippon Seisen (5659)	Shin Nippon Denko (5563)
Nonferrous Metals	Showa Denko HD (5805)	Asaka Riken (5724)
Metal Products	Bunka Shutter (5930)	LIXIL (5938)
Machinery	Disco (6146)	Tsugami (6101)
Electronic Equipment	Fuji Electric (6504)	Ibiden (4062)
Transport Equipment	Shimano (7309)	Japan Engine Corporation (6106)
Precision Equipment	Shimadzu (7701)	HOYA (7741)
Other Products	Snow Peak (7816)	Maeda Kosen (7821)
Electricity and Gas	EREX (9517)	Shizuoka Gas (9543)
Land Transportation	Hitachi Transport System (9086)	Senko Group HD (9069)
Marine Transportation	Nippon Yusen KK (9101)	Iino Kaiun Kaisha (9119)
Air Transportation	ANA HD (9202)	Japan Airlines (9201)
Warehousing and Transportation	Mitsubishi Logistics Corporation (9301)	MITSUI-SOKO (9302)
Information and Communication	GMO Payment Gateway (3769)	MoneyForward (3994)
Wholesale business	Tomen Devices (2737)	Matsuda Sangyo (7456)
Retailing	ZOZO (3092)	Golf Digest Online (3319)
Banking	Tohoku Bank (8349)	Bank of Kyoto (8369)
Securities and Commodity Futures	JAFCO Group (8595)	Monex Group (8698)
Insurance	SOMPO HD (8630)	MS&AD Insurance Group HD (8725)
Other Financial Services	Japan Securities Finance (8511)	Aiful Corporation (8515)
Real Estates	Nomura Real Estate HD (3231)	Mitsubishi Estate (8802)
Service	Japan M&A Center HD (2127)	Mixi (2121)
